# Greater impact of dietary fat manipulation than apolipoprotein E genotype on *ex vivo* cytokine production – Insights from the SATgenε study^☆^

**DOI:** 10.1016/j.cyto.2013.12.015

**Published:** 2014-04

**Authors:** Athanasios Koutsos, Kim G. Jackson, Stacey Lockyer, Andrew Carvalho-Wells, Anne M. Minihane, Julie A. Lovegrove

**Affiliations:** Hugh Sinclair Unit of Human Nutrition, Institute for Cardiovascular and Metabolic Research (ICMR), Department of Food and Nutritional Sciences, University of Reading, Reading RG6 6AP, UK

**Keywords:** APOE, apolipoprotein E, BMI, body mass index, CHO, carbohydrate, CRP, C-reactive protein, CVD, cardiovascular disease, DHA, docosahexaenoic acid, GM-CSF, granulocyte-macrophage colony-stimulating factor, HSF, high saturated fat, ICAM-1, inter-cellular adhesion molecule-1, IFN, interferon, IL, interleukin, LDL-C, low density lipoprotein-cholesterol, LF, low fat, LPS, lipopolysaccharide, MUFA, monounsaturated fatty acid, NF-κB, nuclear factor kappa B, PUFA, polyunsaturated fatty acid, SFA, saturated fatty acid, SNP, single nucleotide polymorphism, TNF-α, tumor necrosis factor-alpha, VCAM-1, vascular cell adhesion molecule, WBC, whole blood culture, *APOE*, Dietary fat manipulation, TNF-α, IL-10, DHA

## Abstract

Apolipoprotein E (*APOE*) genotype is believed to play an important role in cardiovascular risk. *APOE4* carriers have been associated with higher blood lipid levels and a more pro-inflammatory state compared with *APOE3*/*E3* individuals. Although dietary fat composition has been considered to modulate the inflammatory state in humans, very little is known about how *APOE* genotype can impact on this response. In a follow-up to the main SATgenε study, we aimed to explore the effects of *APOE* genotype, as well as, dietary fat manipulation on *ex vivo* cytokine production. Blood samples were collected from a subset of SATgenε participants (*n* = 52/88), prospectively recruited according to *APOE* genotype (*n* = 26 E3/E3 and *n* = 26 E3/E4) after low-fat (LF), high saturated fat (HSF) and HSF with 3.45 g docosahexaenoic acid (DHA) dietary periods (each diet eight weeks in duration assigned in the same order) for the measurement of *ex vivo* cytokine production using whole blood culture (WBC). Concentrations of IL-1beta, IL-6, IL-8, IL-10 and TNF-alpha were measured in WBC supernatant samples after stimulation for 24 h with either 0.05 or 1 μg/ml of bacterial lipopolysaccharide (LPS). Cytokine levels were not influenced by genotype, whereas, dietary fat manipulation had a significant impact on TNF-α and IL-10 production; TNF-α concentration was higher after consumption of the HSF diet compared with baseline and the LF diet (*P* < 0.05), whereas, IL-10 concentration was higher after the LF diet compared with baseline (*P* < 0.05). In conclusion, our study has revealed the amount and type of dietary fat can significantly modulate the production of TNF-α and IL-10 by *ex vivo* LPS-stimulated WBC samples obtained from normolipidaemic subjects.

## Highlights

• The effect of diet and genotype on inflammatory response was explored in humans.
• Cytokine production was not affected by apolipoprotein E (*APOE*) genotype.
• TNF-α concentration significantly increased after a high saturated fat diet.
• IL-10 concentration was significantly higher after a low fat diet.
• The amount and type of dietary fat modulated cytokine production.

## 1 Introduction

The aetiology and progression of cardiovascular disease (CVD) is affected by both environmental and genetic factors [1]. The most widely researched common gene variants, with respect to CVD risk are the apolipoprotein E (*APOE*) single nucleotide polymorphisms (SNP) [2]*.* Although the literature is not fully consistent [3,4] *APOE4* carriers (approximately 25% of the Caucasian population), have been reported to have a higher risk of CVD. This was originally attributed to elevated blood lipid levels in this subgroup [4]. However, the mechanisms that relate *APOE4* to increased CVD risk may be more complex than solely a lipid effect [1,2] with studies largely conducted in transgenic animals or cell lines indicating that the *APOE4* allele is related to a more pro-oxidative and pro-inflammatory state compared with the *APOE3* allele [1,2].

Dietary fat manipulation may play an important role in the inflammatory response [5,6]; several studies indicated increasing levels of pro-inflammatory cytokines after a high fat meal or chronic consumption of a high fat diet [5,7,8]. However, few studies have focused on the impact of dietary fat composition on these markers in response to iso-energetic diets. Recently, we have reported that *APOE* genotype influences the C-reactive protein (CRP) response to dietary fat intake, with higher concentrations after diets rich in saturated fat (HSF) and HSF with 3.45 g/d of docosahexaenoic acid (DHA) in *APOE4* carriers, relative to a low fat (LF) diet [9]. Yet, there are no studies investigating the combined effect of *APOE* genotype and fat manipulation on cytokine production in normolipidaemic subjects.

The aim of the present study was to investigate the effect of these three iso-energetic diets differing in fat quantity and quality on *ex vivo* whole blood culture (WBC) cytokine production according to *APOE* genotype. The WBC technique measures cytokine production following a pro-inflammatory stimulant and is considered a more physiologically meaningful and informative measure of inflammatory status in humans relative to the more traditional assessment of plasma cytokines in fasting blood.

## 2 Material and methods

### 2.1 Subjects and study design

A subset of the normolipidaemic participants from the SATgenε study (*n* = 52/88), who were prospectively recruited according to *APOE* genotype (*n* = 26 E3/E3 and *n* = 26 E3/E4) provided blood samples at the beginning of the study (baseline) and eight weeks after the low fat (LF), high saturated fat (HSF) and HSF with 3.45 g/day DHA (HSF-DHA) diet for the determination of *ex vivo* cytokine production using whole blood culture. The target macronutrient composition of the diets are shown in Table 1 and a detailed description of study design and dietary manipulation are presented in Carvalho-Wells et al. [9].

### 2.2 Stimulation of whole blood cultures

Blood samples collected in EDTA tubes were diluted 1:9 with RPMI 1640 medium (Sigma, UK) supplemented with 1% antibiotics, 1% l-glutamine and 1% non-essential amino acids (BioScience, UK). Subsequently, the diluted blood sample was cultured in 12-well plates (Greiner bio-one, UK), with 10 or 0.5 μg/ml of bacterial lipopolysaccharide (LPS) (*E. coli* 026:B6, Sigma, UK), leading to a final concentration of 1 or 0.05 μg/ml, respectively. Cultures were incubated at 37 °C for 24 h before centrifugation at 700×*g* to isolate the supernatant, which was stored at −20 °C until analysis. The monocyte count of each sample was measured by the Pathology Department at the Royal Berkshire Hospital in Reading.

### 2.3 Measurement of the cytokine concentration using the Luminex method

A Human Cytokine 10-Plex Panel (IL-1β, IL-2, IL-4, IL-5, IL-6, IL-8, IL-10, IFN-γ, TNF-α, and GM-CSF; Invitrogen, Life Technologies) was used to measure the concentration of cytokines in the whole blood culture supernatant using the Luminex 200. Only IL-1β, IL-6, IL-8, IL-10 and TNF-α were detectable in the whole blood culture samples. Cytokine production was expressed as μg/10^3^ monocytes as previously reported by Nagata et al. [10] and Rohleder et al. [11].

### 2.4 Statistical analysis

A one-within, one-between repeated measures ANOVA was used to analyse the effects of the different diets on the whole blood culture cytokine concentrations in the two genotype groups. Logarithmic or square-root transformation was applied to the variables that were not normally distributed. When statistical differences were found, data were further tested by the least significant difference (LSD) post hoc test. The statistical analysis was performed using SPSS version 17.0 (Statistical Package for Social Sciences, SPSS Inc., Chicago, Illinois, USA). *P* ⩽ 0.05 was considered statistically significant.

## 3 Results

**Fig. 1:**
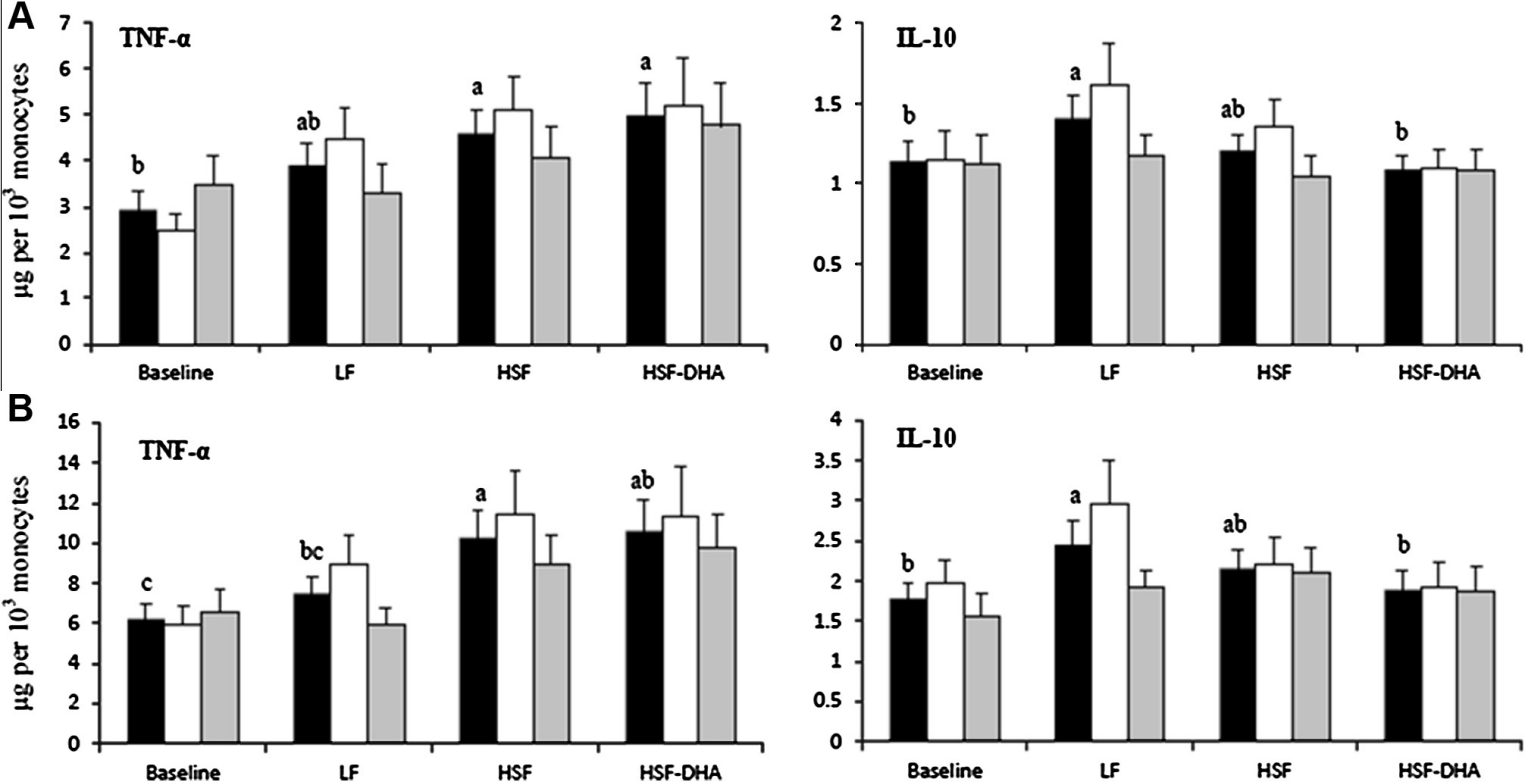
Effect of dietary fat manipulation and genotype on TNF-α and IL-10 production (μg per 10^3^ monocytes) following stimulation of whole blood cultures with (A) 0.05 μg/ml and (B) 1 μg/ml of lipopolysaccharide (LPS) for 24 h. Values are means for all the subjects (black bars, *n* = 50), E3/E3 (white bars, *n* = 26), E3/E4 (grey bars, *n* = 24), with their standard errors represented by vertical bars. An overall diet effect was evident for TNF-α (*P* = 0.012, 0.006) and IL-10 (*P* = 0.036, 0.049) at 0.05 and 1 μg/ml of LPS respectively, when the genotype groups were combined (*n* = 50). ^a,b,c^ Mean values with unlike superscript letters were significantly different (*P* ⩽ 0.05), after a post hoc analysis. *Abbreviations:* LF, low fat diet; HSF, high saturated fat diet, HSF-DHA, HSF diet with 3.45 g/d of docosahexaenoic acid.

Two subjects (*APOE4* carriers) were excluded because of missing monocyte population data. Cytokine production by either 0.05 μg/ml or 1 μg/ml LPS-stimulated WBC was not significantly affected by genotype (Table 2, Fig. 1). In the data for the two genotype groups combined, dietary fat manipulation significantly affected TNF-α and IL-10 production, for both 0.05 μg/ml (*P* = 0.012 and *P* = 0.036, respectively, repeated measures ANOVA) and 1 μg/ml of LPS (*P* = 0.006 and *P* = 0.049, respectively, repeated measures ANOVA) (Fig. 1). Post hoc analysis revealed that TNF-α production was significantly higher in the WBC supernatant after the subjects consumed the HSF diet compared with baseline (*P* = 0.004 and *P* = 0.002, for 0.05 and 1 μg/ml LPS, respectively) and the LF diet (*P* = 0.020, for 1 μg/ml LPS only) (Fig. 1). The HSF-DHA diet resulted in higher TNF-α concentration in comparison to baseline (*P* = 0.012 and *P* = 0.021, for 0.05 and 1 μg/ml LPS, respectively) (Fig. 1). For both LPS concentrations, the consumption of the LF diet resulted in significantly higher concentrations of IL-10, compared with baseline (*P* = 0.013 and *P* = 0.015, for 0.05 and 1 μg/ml LPS, respectively) and the HSF-DHA diet (*P* = 0.026 and *P* = 0.050, for 0.05 and 1 μg/ml LPS, respectively) (Fig. 1). The levels of IL-1β, IL-6 and IL-8 were not significantly modulated by dietary fat composition (Table 2).

## 4 Discussion

The present study investigated the impact of *APOE* genotype on the *ex vivo* cytokine response of normolipidaemic subjects to chronic dietary fat manipulation. It has been indicated that *APOE* has immuno-modulatory properties that could affect the risk of CVD [2]. *APOE* genotype has been reported to affect macrophage cytokine secretion [1,2] and, thus, may be predicted to influence inflammatory responses. As we previously reviewed [2] animal and cell culture models and limited human study evidence showed that pro-inflammatory cytokine levels, such as TNF-α and IL-6, were higher whereas the anti-inflammatory cytokine IL-10 concentrations were lower in *APOE4*-expressing cells or genotype groups compared to *APOE3* homozygotes. In contrast, in the current study, cytokine production was not significantly affected by *APOE* genotype suggesting that in normolipidaemic adults *APOE* genotype may not be a significant determinant of the circulating pro-inflammatory cytokine status [2].

Several observational and intervention studies have explored the impact of a high fat diet, as well as high SFA diet, on pro-inflammatory markers [5–8]. Nappo et al. [7] have reported that a high fat meal (59% fat) increased the postprandial levels of TNF-α, IL-6, intercellular adhesion molecule-1 (ICAM-1) and vascular cell adhesion molecule-1 (VCAM-1) in normal subjects, whereas an isoenergetic high carbohydrate meal had no effect. Longer-term dietary studies have also reported a significant effect of dietary fat manipulation on the inflammatory response. Baer et al. [8] have shown that the consumption of specific saturated fatty acids including lauric, myristic, palmitic and stearic acid for a five-week period was associated with higher levels of CRP, fibrinogen and IL-6. These results are in agreement with our data, where TNF-α, an important pro-inflammatory cytokine involved in the progression of atherosclerosis, significantly increased after consumption of a diet high in SFA (HSF). Previous analysis of our data has also shown that the HSF diet resulted in a significant increase in fasted serum CRP, compared with the LF diet [9]. These data suggests that the deleterious impact of habitual high fat and high saturated fat intake may in part be mediated via an impact on TNF-α production.

Consumption of the LF diet was associated with a significant increase in IL-10 concentrations compared with the baseline. IL-10 is an anti-inflammatory cytokine, which down-regulates many inflammatory pathways that are associated with atherosclerosis [12]. In particular, it inhibits the production of NF-κB and thus, suppresses cytokine production [12]. Moreover, it is associated with beneficial effects in patients with acute coronary disease [13]. To the best of our knowledge, this study is among the first to evaluate the effect of different iso-energetic fat diets on the *ex vivo* levels of an anti-inflammatory cytokine, such as IL-10 in WBC samples obtained from human subjects. With reference to the supplementation of the HSF diet with long chain n-3 fatty acids (3.45 g/d of DHA) we did not observe any significant changes in cytokine levels between *APOE3/E3* and *APOE4* carriers nor in our study group as a whole. Although long-chain n-3 fatty acids are purported to be anti-inflammatory, with for example demonstrated benefits on plaque inflammation [14] and the need for use of non-steroidal anti-inflammatory drugs in rheumatoid arthritis patients [15], our results are in general agreement with the majority of human intervention studies, including our main SATgenε study, which have not shown a clear effect on circulating inflammatory status [16].

In this study two concentrations of LPS were used for stimulation of WBC (1 μg/ml and 0.05 μg/ml) with both giving comparable results. It is therefore suggested that in future studies it would be advisable to use the more physiological concentration of 0.05 μg/ml. Moreover, since monocytes are the main site of cytokine production after LPS stimulation in whole blood, a correction with monocytes is considered by many to be the most appropriate method to use [10,11].

In conclusion, dietary fat composition but not *APOE* genotype significantly influenced *ex vivo* pro- and anti-inflammatory cytokine production after LPS stimulation of WBC samples obtained from normolipidaemic adults.

## Figures and Tables

**Table 1:** Target daily macronutrient composition of the 3 isoenergetic diets.

Target macronutrient composition	LF diet	HSF diet	HSF-DHA diet
Energy from fat (%)	24	38	38
SFA (%)	8	18	18
MUFA (%)	8	12	12
PUFA (%)	6	6	6
Energy from carbohydrates (%)	59	45	45

*Abbreviations:* LF, low fat diet; HSF, high saturated fat diet; HSF-DHA, high saturated fat diet with 3.45 g/d docosahexaenoic acid (DHA); SFA, saturated fat; MUFA, monounsaturated fatty acids; PUFA, polyunsaturated fatty acids; %, % of the total energy intake.

**Table 2:** Effect of dietary fat manipulation and genotype on cytokine production (μg per 10^3^ monocytes) following stimulation of whole blood cultures with 0.05 and 1 μg/ml lipopolysaccharide for 24 h.

Cytokine	Baseline		LF diet		HSF diet		HSF-DHA diet		ANOVA		
	Mean	SEM	Mean	SEM	Mean	SEM	Mean	SEM	Diet (*P* value)	Genotype (*P* value)	Diet *x* genotype (*P* value)
*0.05 μg/ml LPS*											
IL-1β									0.100	0.217	0.652
E3/E3	9.49	0.97	12.96	1.45	11.75	1.22	11.43	1.71			
E3/E4	9.43	1.18	9.91	1.26	10.31	1.21	8.72	1.31			
All	9.46	0.75	11.50	0.98	11.06	0.86	10.13	1.09			

IL-6									0.162	0.091	0.492
E3/E3	51.1	5.3	77.2	13.2	61.0	6.5	55.4	7.7			
E3/E4	46.5	6.0	46.5	4.1	49.9	6.0	49.3	8.1			
All	48.9	4.0	62.5	7.4	55.7	4.5	52.5	5.5			

IL-8									0.089	0.782	0.717
E3/E3	378.7	63.5	434.5	49.4	460.5	60.6	335.9	42.9			
E3/E4	382.4	69.6	475.3	60.9	374.6	64.0	360.3	56.1			
All	380.5	46.5	454.1	38.6	419.3	44.0	347.6	34.6			
											
*1 μg/ml LPS*											
IL-1β									0.084	0.299	0.607
E3/E3	13.68	1.16	16.13	1.61	15.55	1.58	15.28	2.32			
E3/E4	12.00	1.44	13.38	1.47	15.81	1.81	12.39	1.71			
All	12.87	0.91	14.81	1.10	15.68	1.18	13.89	1.46			

IL-6									0.139	0.115	0.734
E3/E3	105.1	13.6	100.4	13.0	122.4	18.5	124.8	29.9			
E3/E4	74.0	11.1	68.7	6.1	116.2	21.1	91.4	14.9			
All	90.2	9.1	85.2	7.6	119.4	13.9	108.8	17.1			

IL-8									0.278	0.983	0.191
E3/E3	592.2	81.4	534.5	66.7	1099.1	520.9	465.2	74.1			
E3/E4	469.1	58.5	698.3	86.0	566	60.7	658.1	163.9			
All	533.1	51.1	613.2	54.6	843.2	272.5	557.8	87.7			

Values represent mean SEM for the data presented according to genotype (E3/E3, *n* = 26 and E3/E4, *n* = 24) and the genotype groups combined (All, *n* = 50). Abbreviations: LPS, lipopolysaccharide; LF, low fat diet; HSF, high saturated fat diet; HSF-DHA, high saturated fat diet with 3.45 g/d docosahexaenoic acid (DHA).
